# Unearthed opium: development of a UHPLC-MS/MS method for the determination of *Papaver somniferum* alkaloids in Daunian vessels

**DOI:** 10.3389/fchem.2023.1238793

**Published:** 2023-07-26

**Authors:** Flaminia Vincenti, Camilla Montesano, Alessandro Ciccola, Ilaria Serafini, Gabriele Favero, Matteo Pallotta, Flavia Pagano, Gaia Di Francesco, Martina Croce, Maria Laura Leone, Italo Maria Muntoni, Manuel Sergi

**Affiliations:** ^1^ Department of Chemistry, Sapienza University of Rome, Rome, Italy; ^2^ Department of Environmental Biology, Sapienza University of Rome, Rome, Italy; ^3^ Department of Public Health and Infectious Disease, Sapienza University of Rome, Rome, Italy; ^4^ Independent Researcher in Archaeology, Foggia, Italy; ^5^ Soprintendenza Archeologia, Belle Arti e Paesaggio per le Province di Barletta—Andria—Trani e Foggia, Foggia, Italy

**Keywords:** opium alkaloids, archeological ceramics, pressurized liquid extraction, mass spectrometry, Daunian

## Abstract

**Introduction:** The analysis of organic residue in ancient vessels to investigate early-age civilization habits is an important archeological application that needs advanced analytical methods. However, these procedures should meet inherent requisites such as low sampling invasiveness and high sensitivity for trace analysis. This study deals with the development of advanced analytical methods for the detection of opium alkaloids in ceramic vessels and its first application to the study of Daunian pots dating back to the VIII–IV sec BC.

**Methods:** All the stages of the analytical procedure, from sampling to analysis, were carefully optimized. Concerning sampling, the traditional scraping approach was compared with a swabbing strategy which permitted minimizing sample encroachment. Extraction was based on pressurized liquid extraction or ultrasound-assisted liquid extraction, followed by dispersive liquid–liquid microextraction, which allowed concentration enrichment. On the other hand, a UHPLC-MS/MS method was specifically developed and validated to obtain reliable data. Some Daunian pots, belonging to the Ceci-Macrini private archeological collection, were selected for sample withdrawal as their iconography could suggest opium usage.

**Results:** Several of the analyzed samples resulted positive to thebaine and less frequently to morphine and codeine; furthermore, 70% of the analyzed items tested positive for at least one opium alkaloid. Positive findings were common to all the samples collected in the pots, suggesting that scraping and swabbing provided comparable results and validating this unusual sampling strategy. All samples were additionally analyzed by UHPLC-HRMS to further improve the confidence level of the identified compounds. The obtained results shed new light on the hypothesis of opium usage by the ancient Daunian civilization. Furthermore, this study provided suitable analytical tools for further investigations on the same topic, with a good level of confidence in the quality of the results.

## 1 Introduction

The peculiar properties of many natural psychoactive plants were well known in antiquity; for example, *Papaver somniferum*, among the most ancient and studied, has been already known since the Sumerian civilization, and the extraction of psychoactive substances, in particular opium, was known by ancient Egyptians and practiced by Cypriots during the Bronze Age, handed down to ancient Greeks, and reached up to the Romans, who reported the first cases of dependence (Nencini, 1997). The use of these substances was mainly linked to medicine and food and, most likely, was part of ritual practices and contact with the afterlife (Kritikos, 1968).

**FIGURE 1:**
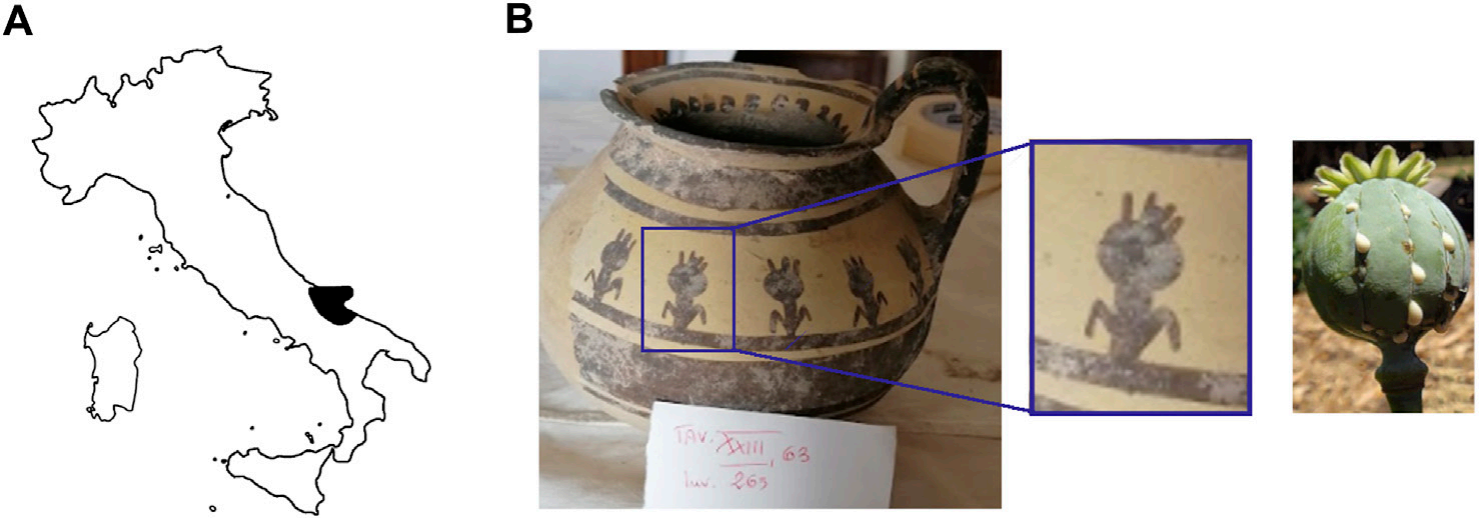
Map showing the localization of Daunia (Apulia IT) **(A)**; magnification of a vessel from the *Ceci-Macrini* collection (ID 265) whose iconography could suggest opium usage; the similarity between the decoration and the poppy capsule is highlighted **(B)**.

A particular case is represented by the Daunians, inhabitants of the historical geographical district of northern Puglia, which, in ancient times, with Peucezia and Messapia, constituted Apulia (Figure 1A). This civilization left us a rich ancient collection of figured stelae and vases belonging to the VIII–IV century BC (Nava, 1998; Mazzei, 2010). These ancient stelae and vases show pictorial and morphological references that could suggest the use of opioids by Daunians, probably in a religious and healing context (Leone, 2002) (Figure 1B).

Similar observations on Cypriote Bronze Age base-ring juglets led some archeologists to propose a link between these vessels and opium trade (Merrillees, 1962), given their distinctive shape, which looks like the capsule of the opium poppy. For a long period, this theory has been controversial, but recently, reliable chemical evidence demonstrated that these vessels once contained opium (Smith et al., 2018; Linares et al., 2022). In several studies, it was shown that opium alkaloids can be detected even in archeological samples through highly sensitive analytical techniques. The first findings were related to morphine (Koschel, 1996), the most abundant alkaloid in opium, but lately, the presence of morphine was questioned because of its instability (Chovanec et al., 2012). Smith et al. reported, most recently, rigorous chemical evidence of more stable opium alkaloids, i.e., papaverine and thebaine, in a sealed British Museum base-ring juglet (Smith et al., 2018), and more recently, Linares et al. published further evidence of the presence of morphinan-based alkaloids in vessels, arising from Tel Yeud burials in Israel, dating to the Late Bronze Age IIA (Linares et al., 2022). All these studies showed that advanced analytical techniques may be a high-level ally for archeologists, thus becoming crucial to answer open archeometric questions; anyway, suitable and robust analytical protocols must be used to produce reliable and incontrovertible results.

The analysis of organic residues absorbed into the ceramic walls of ancient vessels in order to determine their past contents is widespread: wine, plant oils and resins, animal fats, etc., are among the most detected compounds (Briggs et al., 2022), also for Daunian archeological contexts (Leone et al., 2015; Muntoni et al., 2021). Sampling protocols, extraction conditions, and detection techniques largely depend on the type of analytes and the objectives of the study (Kałużna-Czaplińska et al., 2016). A major distinction can be drawn between noninvasive and destructive methods, taking into account that often, it is not possible to sample an archeological remain and when possible, the sample must be considered unique and unrepeatable. Several spectroscopic techniques (mainly Raman and Fourier-transform infrared spectroscopies) fall in the first group, but the advantage of being nondestructive is balanced by the limited confidence level for analyte identification. For this reason, mass spectrometric (MS) techniques are generally preferred (Rosiak et al., 2020; Cank et al., 2021) and may be coupled with separative techniques, i.e., gas chromatography (GC) and liquid chromatography (LC). Both GC-MS (Kałużna-Czaplińska et al., 2016) and LC-MS possess numerous benefits for the analysis of chemical residues and can be used to separate complex mixtures, determine trace levels of organic residues, and/or perform quantitative experiments; however, they generally require extensive sample preparation.

The analysis of ancient pottery generally involves the withdrawal of several samples from a relevant number of vessels (Irto et al., 2022), whole potsherds, or more frequently, small amounts of material can be collected by scraping the interior surfaces of the pots (Sanjurjo-Sánchez et al., 2018). The collected residues represent very complex matrices; therefore, it is necessary to perform an adequate sample treatment to purify and isolate the target compounds. The literature data about alkaloid extraction show that solvent-based extractions are the most common. Smith et al. extracted the target analytes by ultrasonication with hydrochloric acid, while solid-phase extraction (SPE) was used for sample clean-up. A mixture of dichloromethane:methanol (2:1; v:v) (Linares et al., 2022) or chloroform:isopropanol (3:1; v:v) (Koschel, 1996) was used for extraction by other authors prior to GC-MS analysis.

The aim of this study was to corroborate the hypothesis of opium usage in ancient Daunia through the analysis of organic residues collected in several Daunian vessels belonging to the private Ceci-Macrini archeological collection (Rossi, 1979). In order to obtain rigorous evidence of the presence of opium alkaloids in these samples, a UHPLC-MS/MS method was specifically developed and validated. Furthermore, sample preparation procedures based on pressurized liquid extraction (PLE) or ultrasound-assisted liquid extraction, together with dispersive liquid–liquid microextraction (dLLME) sample enrichment, were exploited to maximize the sensitivity of the method. Two kinds of sampling approaches were carried out: scraping was compared with a less-invasive swabbing approach using commercially available wipes.

## 2 Materials and methods

### 2.1 Standards and reagents

The standards of morphine, codeine, and thebaine were supplied by LGC Standard (Milan, Italy) in methanol solution at 1 mg mL^−1^; internal standard morphine-d_3_ was purchased from the same company as a crystalline powder with a 98% degree of purity; IS working solution (IS-WS) was prepared in methanol at 100 ng mL^−1^.

A standard working mixture containing the three analytes at 1 μg mL^−1^ in methanol was obtained by the appropriate dilution of the standards and was stored at −20°C.

Water, methanol, acetonitrile, 2-propanol, and chloroform, all LC-MS grade, were supplied by Fisher Chemical (Rodano (MI), Italy). Formic acid was provided by VWR Chemicals (Milan, Italy). Cetyltrimethylammonium bromide (CTAB), sodium carbonate, sodium bicarbonate, and ammonium formate were purchased from Merck Life Science S.r.l. (Milan, Italy). A 25 mM CTAB solution in water was prepared for PLE.

### 2.2 Sample collection

Different samples from the 14 Daunian pots belonging to the Ceci-Macrini private collection located in Andria (Apulia, IT) were collected as reported in Table 1. The sampling protocol was performed in agreement with the Italian Ministry of Culture (MiC)—Soprintendenza Archeologia, Belle Arti e Paesaggio per le Province di Barletta-Andria-Trani e Foggia.

**TABLE 1:** List of the analyzed vessels, with photographs, description, and dating information. For each vessel, several samples were collected and labeled on the basis of the sample strategy ((S)—scraping; (AP)—Alco-prep; and (PE) and (PW)—cotton pads soaked with ethanol and water, respectively.).

Sample ID	Photograph	Description	Dating	Sampling			
				Alco-prep (AP)	Cotton pads- H_2_O (PW)	Cotton pads- EtOH (PE)	Scraping (S)
205	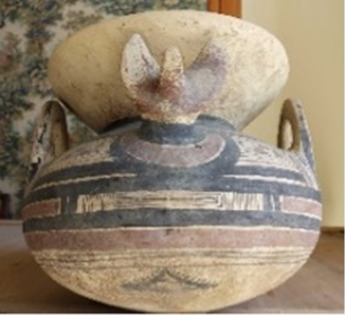	Olla with two-tone decoration, with expanded lip and zoomorphic protomes	Second half of the VI sec BC	205-AP1	205-PW1	205-PE1	
				205-AP2			
211	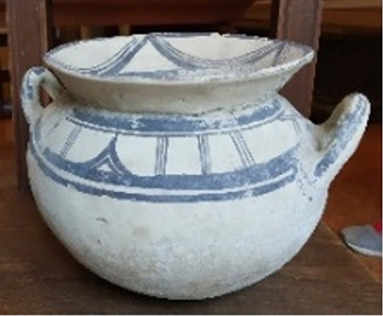	Olla with monochrome decoration	Between mid-VI and half IV sec BC	211-AP1	211-PW1	211-PE1	211-S1
							211-S2
							211-S3
263	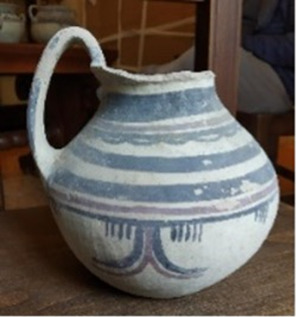	Jug with two-tone decoration	Late V sec BC	263-AP1	263-PW1	263-PE1	263-S1
				263-AP2	263-PW2		
265	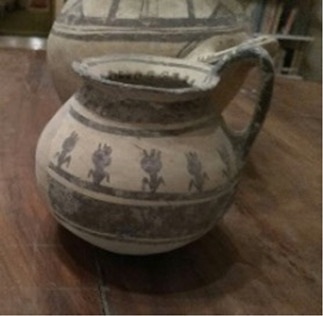	Jug decorated with opium poppy capsules	V sec BC	265-AP1	265-PW1	265-PE1	265-S1
				265-AP2	265-PW2		265-S2
				265-AP3			265-S3
274	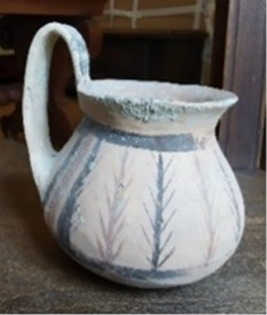	Jug decorated with plants with lanceolate leaves	V sec. AC	274-AP1			274-SList1
				274-AP2			274-S2
316	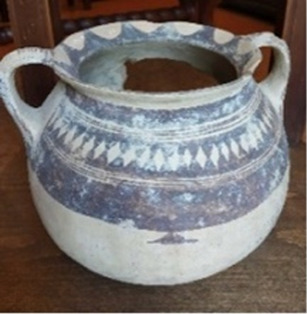	Olla with monochrome decoration	*Protodaunio IX–VIII sec BC*	316-AP1	316-PW1	316-PE1	316-S1
				316-AP2			
317	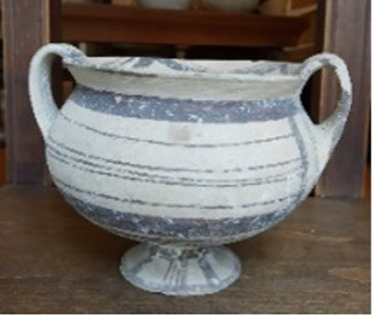	Enotrio-peuceta olla with monochrome decoration	Between the end of the VII and mid-VI sec BC	317-AP1			317-S1
				317-AP2			
333	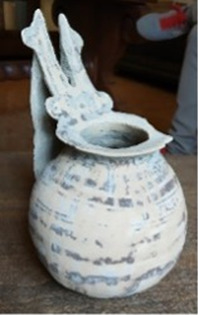	Jug with monochrome decoration and forked handle	End of the VIII and all the VII sec BC	333-AP1			333-S1
				333-AP2			
337	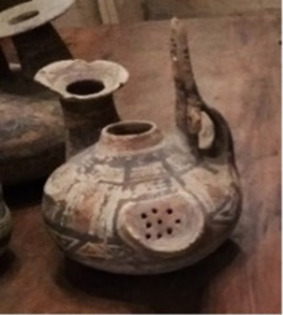	Vase-filter with two-tone decoration	VI sec BC	337-AP1	337-PW1	337-PE1	337-S1
				337-AP2	337-PW2		
				337-AP3			
				337-AP4			
341	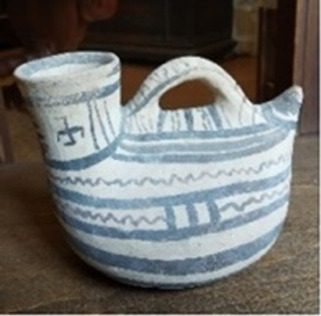	Askos with monochrome decoration	VI–V sec BC	341-AP1			341-S1
346	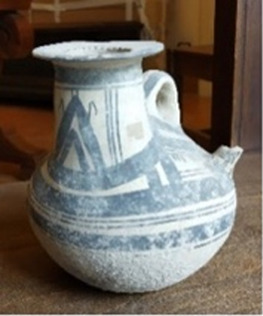	Askos with monochrome decoration	End VI and all V sec BC	346-AP1			346-S1
				346-AP2			
417	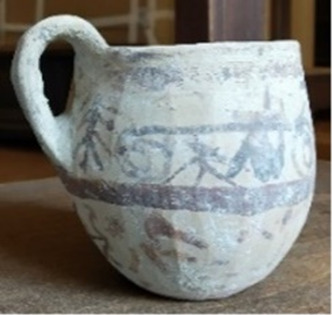	Indigenous cup with two-tone decoration	VI–V sec BC	417-AP1			417-S1
				417-AP2			
455	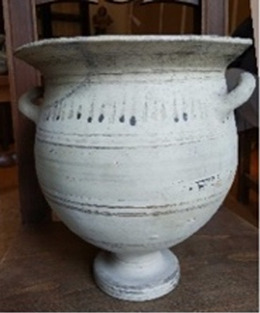	Column crater	IV–III sec BC	455-AP1	455-PW1	455-PE1	455-S1
				455-AP2			
533	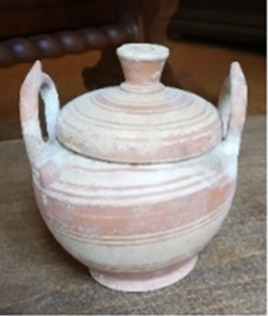	Stamnos with monochrome decoration	Last third IV sec BC	533-AP1			533-S1

Different sampling techniques were implemented; in all cases, the samples were obtained in minimally invasive procedures, considering the historical, artistic, and cultural value of the vessels. To prevent exogenous contamination through hands, nitrile gloves were always worn during the handling of the vessels. First, different samples were obtained by swabbing the interior surface of the vessels with an Alco-prep^®^ (a ready-to-use swab saturated with 70% 2-propanol) and, if allowed by the sampling surface, also with cotton pads soaked in ethanol or water. In addition, if visible debris (ground, ceramic, etc.) were found in the pot, an aliquot (200–500 mg) of the residue was collected by scraping with a disposable scalpel the interior surface of the vessel. The collected samples were labeled as follows: (S)—scraping; (AP)—Alco-prep; and (PE) and (PW)—cotton pads soaked with ethanol and water, respectively. A total of 63 samples were collected.

To avoid cross-contamination, all samples were collected in separate Eppendorf tubes, sealed, and sent to the laboratory of Sapienza University of Rome; the samples were treated separately.

### 2.3 Sample preparation

#### 2.3.1 (S) sample extraction

Alkaloid extraction for (S) samples was performed by PLE. The instrument used was an ASE 200 Accelerated Solvent Extractor from Dionex (Sunnyvale, CA, United States) equipped with 24 stainless steel extraction cells of 1 mL.

An aliquot of 100 mg of each sample was homogenized with diatomaceous earth as the supporting material; the mixture, spiked with 10 μL of IS-WS at 100 ng mL^-1^, was used to fill the extraction cell which was secured at the top and bottom with cellulose filters. Then, the cell was loaded into the instrument. The extracting solvent consisted of a mixture of 0.25 mM CTAB:2-propanol (80:20; v:v). A single extraction cycle was performed with the following parameters: T = 150°C, *p* = 1,450 psi, preheat time = 2 min, heat time = 7 min, static time = 5 min, and purge time = 1 min. About 7.5 mL of the extract was collected for each sample in a separate 50 mL vial sealed with PTFE solvent-resistant septa. The extract was transferred into a 15 mL conical tube and centrifuged at 2,600 *g* for 5 min to promote the sedimentation of all possible solid residues; the supernatant was collected in a separate conical tube.

#### 2.3.2 (AP), (PE), and (PW) sample extraction

All swab types were extracted by in syringe ultrasound-assisted extraction (iS-UAE), a technique specifically developed for this purpose. In brief, following the removal of the plunger, the swab was transferred in a disposable 10 mL syringe, parafilm-sealed at the luer lock; 1 mL of 2-propanol containing the IS-WS at 10 ng mL^-1^ was added directly into the syringe. The plunger was then loosely reassembled, and the syringe was vortex-shaken for 30 s and then immersed for 10 min in an ultrasonic bath thermostated at 25°C. By squeezing the swab using the syringe plunger, the extract was collected in a 15 mL conical tube and centrifuged as described for (S) samples. The supernatant was added to a conical tube containing 6.5 mL of a 0.25 mM CTAB solution.

### 2.4 Clean-up

Sample clean-up was carried out by dLLME. For each sample, the supernatant, obtained as previously described, was added with 0.7 mg of NaCl and 1 mL of carbonate buffer at pH 9.5. Once homogenized, 0.5 mL of 2-propanol (disperser solvent) and 0.2 mL of chloroform (extraction solvent) were rapidly injected into the solution. The obtained sample was vortex-mixed for 30 s, sonicated at 25°C for 10 min, and then, centrifuged at 2,600 *g* for 5 min at 4°C to promote chloroform sedimentation. The latter was collected, evaporated under a gentle nitrogen flow, and finally, reconstituted with 100 μL of water:methanol (50:50; v:v). 3 μL was injected into the UHPLC system.

### 2.5 UHPLC-MS/MS analysis

The UHPLC-MS/MS system consisted of an ExionLC AD System from SCIEX (Framingham, MA, United States) coupled with a QTRAP 6500+, hybrid quadrupole-linear ion trap mass spectrometer from SCIEX, equipped with an ESI source, operating in the positive ion mode. Data acquired were elaborated by using SCIEX OS software (version 2.0.1.).

Analyte separation was performed using an Acquity UPLC BEH C18 (100 × 2.1 mm i.d., packed with 1.7 µm particles) from Waters. The column was maintained at 40°C. Mobile phases were 5 mM ammonium formate (phase A) and 0.1% HCOOH in MeCN:MeOH (75:25; v:v) (phase B), and the flow was 400 μL min^-1^. The elution gradient was structured as follows: phase B was maintained at 0% for 0.5 min and then increased to 20% in 2 min, to 30% in 3.1 min, to 40% in 2 min, then to 50% in 2.5 min, and finally, to 100% in 1.1 min. This condition was maintained for 2 min. The total chromatographic run time, including the equilibration stage between two different analyses, was 16.5 min.

QTRAP source parameters were set as follows: curtain gas 30 units, ion source gas 1 and 2 at 55 and 60 units, respectively, ionspray voltage 5500 V, and source temperature 450°C.

To perform targeted identification, increasing compound identification confidence at the same time, an information-dependent acquisition (IDA) approach was developed: multiple reaction monitoring (MRM) was used as the survey scan, while enhanced product ion (EPI) was selected as the dependent scan. For morphine, thebaine, and codeine, for which standards were available, all parameters were optimized by direct infusion of the relative standards; for the other compounds of interest, MRM transitions were inferred from the literature data (Mueller et al., 2005; Nesměrák et al., 2019). The IDA criteria were set to conduct EPI scans for each detected MRM transition which exceeded 5,000 cps (selected threshold). The five most intense signals were selected during every scan. This mode has the advantage of providing a concomitant product ion spectrum when the required conditions are fulfilled. The EPI scan range was between 50 and 450 Da, while the scan rate was 10,000 Da s^-1^. All spectra obtained were subsequently matched with the commercial library mzCloud and an in-house library. MS parameters are listed in Table 2.

**TABLE 2:** MRM method parameters..

Analyte	Rt	Q1	DP	EP	Q3	CE	CXP
Morphine	3.4	286.0	115	7	**152.4**	76	12
					165.3	50	14
Morphine-d3	3.4	289.0	115	7	**165.0**	50	14
Codeine	3.7	300.1	81	5.5	**166.0**	52	20
					154.3	55	20
Thebaine	5.6	312.1	56	10	**58.0**	43	8
					251.1	37	20
*Papaverine*		*340.1*	*85*	*10*	*324.0*	*35 ± 15*	*10*
					*296.0*	*35 ± 15*	*10*
*Noscapine*		*414.1*	*85*		*353.0*	*35 ± 15*	*10*
					*323.0*	*35 ± 15*	*10*
*Cotarnine*		*238.1*	*85*	*10*	*220.0*	*35 ± 15*	*10*
					*205.0*	*35 ± 15*	*10*

Parameters inferred from the literature are listed in italics. Quantifier ions are highlighted in bold (Rt: retention time; Q1: precursor ion mass; DP: declustering potential; EP: entrance potential; Q3: product ion mass; CE: collision energy; CXP: cell exit potential).

### 2.6 UHPLC-HRMS/MS analysis

High-resolution mass spectrometry (HRMS) was used for untargeted analysis and to further improve the confidence level of the identified compounds. The UHPLC equipment consisted of a Dionex UltiMate^®^ 3000 RSLC system (Thermo Fisher Scientific, San Jose, CA) comprising an auto-sampler equipped with a 100 μL loop and vacuum degasser. Analyte identification was performed on a Q-Exactive Orbitrap (Thermo Fisher Scientific, Bremen, Germany) equipped with a heated electrospray ionization source (H-ESI) operating in both negative and positive ion modes. Untargeted analyses were conducted according to a previously published UHPLC-HRMS/MS method (Vincenti et al., 2020). In brief, analyses were carried out in a full-scan/data-dependent (full MS/dd-MS2) acquisition mode, with an inclusion list to preferentially select specific ions for the MS/MS scan, as listed in Table 3. The Q-Exactive resolution was set at 35,000 FWHM for full-scan analyses, while MS/MS experiments were carried out at a resolution of 17,500 FWHM. Three different normalized collision energies were applied to obtain the MS/MS spectra, namely, 10, 30, and 50.

**TABLE 3:** Inclusion list of precursor ions for HRMS ddMS^2^ analysis.

Analyte	Formula	Mass [m/z]
3-Methoxycinnamic acid	C_10_H_10_O_3_	177.0551
Apomorphine	C_17_H_16_NO_2_	268.1337
Atropine	C_17_H_23_NO_3_	290.1751
Codeine	C_18_H_21_NO_3_	300.1594
Cotarnine	C_12_H_16_NO_4_	238.1074
Dehydrogen hydrocotarnine	C_12_H_14_NO_3_	220.0968
Hydrocotarnine	C_12_H_16_NO_3_	222.1122
Hyoscyamine	C_17_H_23_NO_3_	290.1751
Meconin	C_10_H_12_O_4_	195.0189
Methylhydrocotarnine	C_13_H_18_NO_3_	236.1281
Morphinan	C_16_H_21_N	228.1752
Morphine	C_17_H_19_NO_3_	286.1438
Narcotine	C_22_H_23_NO_7_	414.1547
Noscapine	C_22_H_23_NO_7_	414.1547
Opianic acid	C_10_H_10_O_5_	209.0450
Oxidimorphine	C_34_H_36_N_2_O_6_	569.2651
Papaveraldine	C_20_H_20_NO_5_	354.1336
Papaverine	C_20_H_21_NO_4_	340.1543
Papaverinol	C_20_H_22_NO_5_	356.1493
Thebaine	C_19_H_21_NO_3_	312.1594

Compound Discoverer 3.3 was used for raw data analysis; a generic workflow for retention time alignment, component detection, and elemental composition prediction was used.

### 2.7 Validation

The proposed UHPLC-MS/MS quantitative method was fully validated for the target analytes, evaluating the following parameters: LOD, LOQ, linearity, selectivity, specificity, IS interference, carryover, accuracy, precision, matrix effect, and recovery.

The LOD was considered as the lowest absolute quantity that provided a signal-to-noise ratio (S/N) for the qualifier ion of 3. For this purpose, five different blank soil samples were collected and pooled together to obtain a representative blank matrix; pooled blank matrix samples were fortified with decreasing quantities of the standard in duplicate, processed as described previously, and finally, analyzed in three different chromatographic runs. For what concerns swabs, decreasing amounts of standards were spiked on a clean surface and sampled. LOQ was determined similarly, and it was considered the lowest quantity of analyte providing S/N = 10 for the qualifier ion. For cotton pads and Alco-prep, the following procedure was used: all swabs were fortified and left to dry to simulate the time elapsed between sampling and analysis.

Linearity was evaluated by preparing six calibration standards in water:methanol (50:50; v:v), and the linearity range was from LOQ to 10 ng for all analytes. The internal standard ratio was used to evaluate the calibration samples analyzed in three different chromatographic runs.

To evaluate method selectivity, three blank samples for each sampling type were processed as previously described, and the absence of any interference at analyte retention time was verified to provide an unambiguous identification of the target analytes. Even the possible interference produced by the presence of morphine-d_3_ was evaluated. To this aim, three samples for each sampling type were fortified with the IS-WS and treated as described previously.

Carryover was evaluated by verifying the absence of any signal above the LOD in all blank samples analyzed after the injection of the highest point of the calibration curve.

Intra-day precision and accuracy were evaluated through the analysis of several QC samples fortified with different quantities of standard, i.e., 0.5, 2.5, and 10 ng. Three different QC samples were prepared for each sample type and for each level. Precision was calculated as the relative standard deviation (RSD%), while accuracy was defined in terms of bias as the relative deviation (%) of the mean calculated quantity compared to the spiked quantity for each level. In both cases, validation parameters were considered acceptable in a range of ±20% for accuracy, while precision was required to be <20% for each analyte.

Matrix effect and recoveries were evaluated at three levels; pooled ground blank matrices, Alco-prep, and cotton pads were fortified with 20 µL of working solutions at 25, 125, and 500 ng mL^-1^. The matrix effect (ME%) due to ion suppression and/or enhancement was calculated for each analyte by comparing the absolute peak area obtained from the analysis of a blank sample spiked after the extraction with the absolute peak area of a reference solution at the same concentration. ME% was considered acceptable in a range of ±20%. Recoveries (R%) were calculated by comparing the absolute peak area of a sample spiked before extraction with the absolute peak area of a blank sample spiked after extraction.

## 3 Results and discussion

### 3.1 UHPLC-MS/MS

As previously described, different mass spectrometric approaches were considered in this study. A QTRAP mass spectrometer was selected to obtain high selectivity and sensitivity, taking into account that, when present, low amounts of opium alkaloids were expected to be found in organic residues from ancient vessels. The MRM-IDA-EPI acquisition mode was selected in order to selectively detect the target analytes while obtaining fragmentation spectra for the detected compounds. This last feature permits increasing the confidence level for compound identification. When analytical standards are available (this was the case for thebaine, codeine, and morphine), this additional information is not crucial; however, for putative MRM transitions, the possibility of matching the spectra with MS/MS libraries can be of utmost importance for compound identity confirmation without standards.

In summary, the MRM-IDA-EPI acquisition mode allowed obtaining several identification points (IPs), i.e., the retention time of the peak, the presence of two overlapping precursor-to-product ion transitions, the ion ratio, and possibly, a complete MS/MS spectrum that can be compared with standards or library spectra. The simultaneous collection of all these IPs led to the confirmation of the analyte’s presence in the sample. Furthermore, this approach may possibly detect additional opium-related compounds, which were not detected by MRM analysis.

### 3.2 UHPLC-HRMS/MS

A Q-exactive high-resolution mass spectrometer was designated for untargeted analysis and to further confirm the identity of the analytes detected in low resolution. Sample acquisition was conducted in full MS/dd-MS2; in these conditions, untargeted full-scan MS was followed by product ion scan of the five most intense peaks. This strategy allowed detecting unexpected compounds that can be putatively assigned based on the exact mass and the isotopic pattern; in addition, the presence of fragmentation spectra for the most intense ions can assist in the process of compound annotation. For analyte identification, the accurate mass of the precursor and fragment ions was taken into account, with a maximum tolerance of 5 ppm. An inclusion list with target opioids, as well as their degradation products (Nesměrák et al., 2019), was incorporated into the method in order to preferentially fragment these target compounds. The proprietary software Compound Discoverer was used to assist untargeted analysis.

The results found in low resolution were confirmed by UHPLC-HRMS/MS; no further analytes were found by untargeted analysis.

### 3.3 Sampling and sample preparation

Sample collection from vessels of archeological significance must be carried out with caution; low invasiveness is the watchword. Sampling of organic residues from ancient pots is typically carried out by scraping the interior surface of the containers. In the present study, scraping was only performed when visible debris were noticeable inside the vessel; this was the case for almost all the samples analyzed (Table 1).

In order to compare this established sampling approach with a less-invasive strategy based on swabbing, the same vessels were sampled with both techniques. To this aim, different types of swabs were investigated: Alco-prep are single-packed, commercially available swabs saturated with 2-propanol; two other solvents were then tested, i.e., water and ethanol, and standard cotton pads were soaked in them. The preliminary results obtained in the laboratory, by spiking the three available opioids on a ceramic mortar, showed that all the tested swabs were effective in collecting them from the surface. The results on real samples are shown in Table 4 and discussed as follows in the § real sample analyses.

**TABLE 4:** UHPLC-MS/MS quantitative results for the analyzed samples. The calculated amount is reported in ng of analyte.

Sample	Sampling	Morphine	Codeine	Thebaine
205	AP1			<LOQ
	AP2			3.5
	PW1			3.7
211	S1			
	AP1			
	PW1			
	PE1			
263	S1			3.4
	AP1			6.6
	AP2			6.4
	PW1			1.3
	PW2			1.8
265	S1			1.3
	S2			1.8
	S3			
	AP1			
	AP2			<LOQ
	AP3	0.8	<LOQ	<LOQ
	PW1	0.7		0.6
	PW2			0.5
	PE1			<LOQ
274	S2			
	AP1			<LOQ
	AP2			<LOQ
316	S1			2.6
	AP1	0.5		2.2
	AP2			
	PW1			
	PE1			
317	S1			
	AP1			
	AP2			
333	S1			
	AP1			
	AP2			
337	S1			
	AP2			
	AP4			
	PW1			
	PE1			
341	S1			2.3
	AP1			<LOQ
346	S1			4.3
	AP1			5.2
	AP2			3.7
417	AP1	1.3		3.2
455	S1			
	AP1			5.8
	AP2			3.3
	PW1			
	PE1			
533	S1			<LOQ
	AP1			<LOQ

#### 3.3.1 dLLME clean-up

**FIGURE 2:**
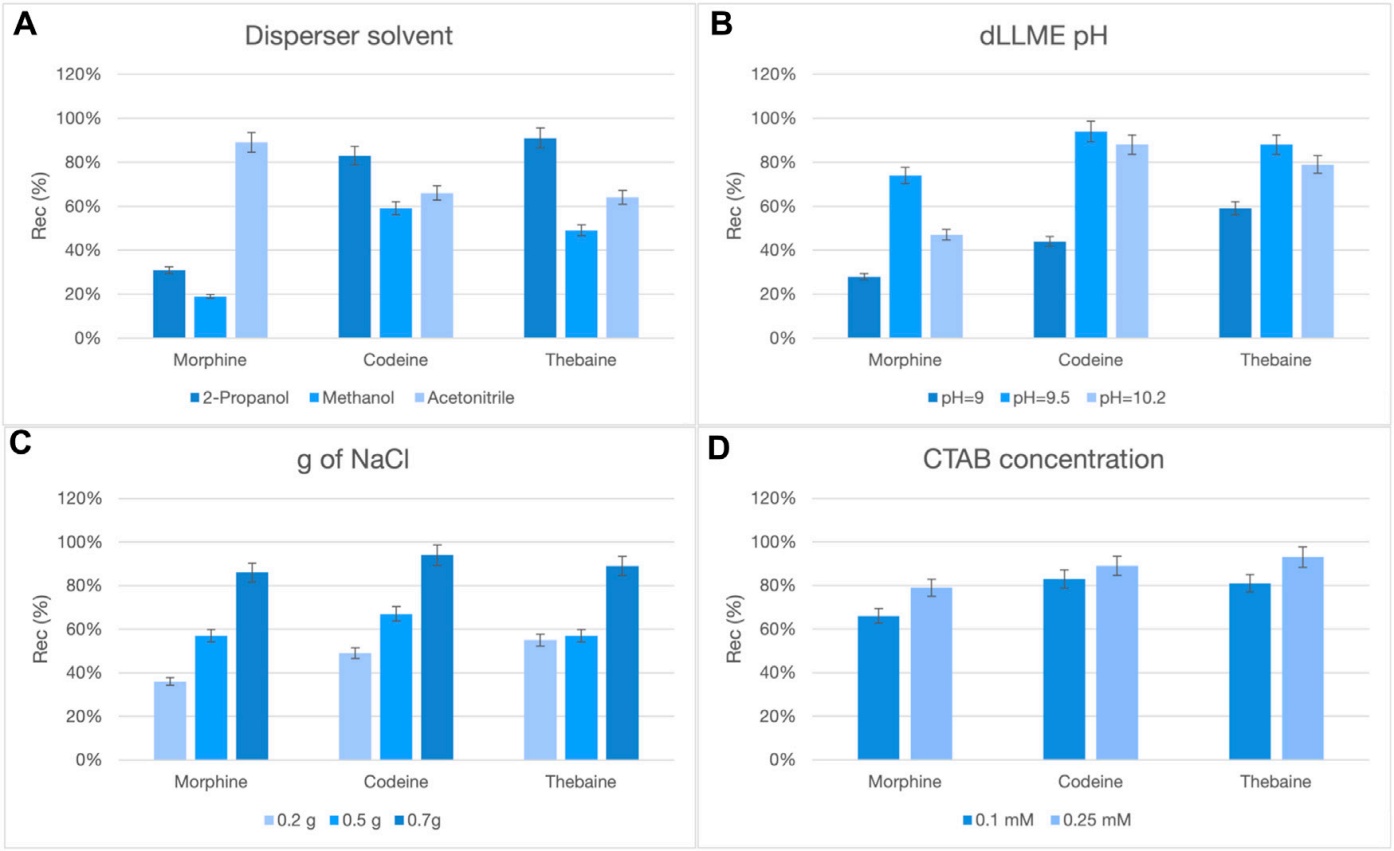
Influence of the dispersing solvent nature **(A)**, pH **(B)**, ionic strength **(C)**, and CTAB concentration **(D)** on dLLME recoveries.

Keeping in mind that a significant enrichment factor would be beneficial, because of the very low concentrations expected, dLLME was selected as the clean-up protocol. This liquid–liquid microextraction technique exploits ternary solvent mixtures to achieve effective extraction of medium-to-high hydrophobic compounds, with decreased amount of solvent and time; high enrichment factors are generally attained. Several parameters were monitored to maximize analyte recovery: the nature of the dispersing solvent, ionic strength, and pH were found to be particularly relevant. In the first attempt, independent of the conditions used, the recovery for morphine was extremely low. An explanation for this behavior is that morphine is charged at almost every pH value because of the co-presence of tertiary amino and phenolic hydroxy groups in its structure, so its partitioning in the organic phase is dampened. The addition of an ion pair reagent, i.e., CTAB, turned out to be a successful strategy (Figure 2); in fact, the positive charge of CTAB may interact with the negatively charged hydroxy group of morphine to promote its partitioning into the organic phase.

#### 3.3.2 PLE and iS-UAE methods

Given that several types of samples were considered in this study, different strategies were developed for analyte extraction. Samples obtained by scraping were extracted by PLE, while swabs were extracted by iS-UAE. This technique was specifically designed to minimize sample handling and avoid the need of cutting the swab into several pieces to insert them in the PLE cell. Regardless of the extraction method and taking into account the complexity and variability of the analyzed samples, sample clean-up was deemed of utmost importance to ensure high quality of the data in terms of reproducibility and ion suppression.

For what concerns (S) sample extraction, PLE was selected. This technique uses solvents at high temperature and pressure to extract analytes from solid or semi-solid samples; because of the high operating temperature and pressure, the organic solvent can be replaced by aqueous solutions, significantly reducing the environmental impact of the extraction procedure. Before loading into the extraction cell, the samples were manually ground and mixed with an inert material (diatomaceous earth) to ensure their uniform dispersion, avoiding aggregation of sample particles and allowing good solvent–sample contact within the extraction cell. In order to find suitable conditions for opioid extraction from a matrix as similar as possible to the sample under investigation, blank soil samples of different types (sandy and clayish) were mixed, and several aliquots were spiked with known amounts of the available standards. Among the several parameters that may influence the PLE recovery rate, the composition of the extraction solvent is of utmost importance. Based on previous studies (Vincenti et al., 2019) and taking into account the subsequent clean-up step, water:2-propanol (90:10, v:v) with 0.25 mM CTAB was tested. This extracting solution yielded good results in terms of recovery (>50% for all the analytes) and was then selected for PLE.

As already mentioned, for (PW), (PE), and (AP) samples, a different protocol was developed. iS-UAE stands for in-syringe ultrasound-assisted extraction; in fact, in this case, the swab is packed in a disposable syringe, and isopropanol extraction is promoted by acoustic cavitation.

### 3.4 Method validation

LODs and LOQs were below 1 ng for all the analytes, confirming that the developed protocols have high sensitivity and are then suitable for opiate residue analysis. Values are reported in Table 5. Linearity was investigated from LOQ to 10 ng, allowing the quantification over several orders of magnitude.

**TABLE 5:** LOD, LOQ, and linearity for the three analytes based on the sampling strategy.

Sampling method	Analyte	R^2	LOD (ng)	LOQ (ng)
S	Morphine	0.9939	0.1	0.2
	Codeine	0.9968	0.05	0.1
	Thebaine	0.9979	0.05	0.1
AP–SW–SE	Morphine	0.9979	0.2	0.5
	Codeine	0.9828	0.2	0.5
	Thebaine	0.9947	0.2	0.5

Selectivity, together with the possible interference of IS, was evaluated by the analysis of several swab and soil blank samples. Three additional blank samples for every sampling type were fortified with morphine-d3. In addition, it must be pointed out that two blank samples, one containing only the IS, were analyzed with every batch of real samples. The absence of any signals belonging to non-deuterated morphine, as well as the absence of every signal of interest, was observed in all the analyzed blanks. These observations showed that the method is selective and the detection of opiates in real samples may not be attributable to the contamination occurred during extraction and/or analysis.

Concerning carryover, all the analyzed blanks did not show any signals at the target analyte retention time, demonstrating that the method was not affected by carryover.

Three different absolute quantities were considered to evaluate precision and accuracy, i.e., 0.5, 2.5, and 10 ng. According to the results listed in Table 6, both extraction protocols yielded optimal results and RSD% was lower than 20%, while bias ranged from −11%–28% for morphine at the lower level tested. The same level was used to verify the matrix effect and recoveries; independent of the extraction protocol, the matrix effect was shown to be not significant, while recoveries were deemed suitable. As expected, because of the use of high temperature and pressure, PLE yielded better recoveries for all the tested analytes compared to iS-UAE (Table 6).

**TABLE 6:** Validation results: precision, accuracy, recoveries, and matrix effects.

Sampling method	Analyte	Precision (%)			Accuracy (%)			Recovery (%)			Matrix effect (%)		
Level		L	M	H	L	M	H	L	M	H	L	M	H
S	Morphine	5	7	3	96	92	99	66	87	96	96	98	105
	Codeine	8	11	2	93	89	102	79	83	95	102	87	96
	Thebaine	3	6	1	94	97	106	85	92	98	98	93	105
AP–SW–SE	Morphine	5	19	9	128	108	99	34	48	48	108	108	104
	Codeine	12	10	11	120	110	110	41	50	57	108	96	97
	Thebaine	5	14	19	95	98	106	67	74	77	94	94	96

### 3.5 Real sample analyses

In this study, several samples collected from different Daunian pots from the Ceci-Macrini collection were analyzed as described previously. A total of 14 pots were selected based on the available archeological information, and special attention was paid to vessels whose iconography could suggest opium usage. An example is shown in Figure 1.

Interestingly, several of the analyzed samples tested positive to thebaine and less frequently to morphine and codeine. Quantitative results expressed as ng of the analyte in the analyzed sample are provided in Table 4, where it can be noted that 10 vessels, or 70% of the analyzed items, tested positive for at least one opium alkaloid. Among the detected analytes, thebaine was the most recurrent, and this finding was not surprising considering that several authors showed that thebaine, together with papaverine, is among the most stable opium alkaloids (Chovanec et al., 2012; Smith et al., 2018). It should also be pointed out that, with the developed method, the recovery was higher for thebaine; this also may have contributed to the prevalent detection of this opioid in the samples. In our samples, neither with low- nor high-resolution mass spectrometry, papaverine was detected, while morphine and codeine, which were known to degrade more rapidly, were observed in some samples. An explanation for these observations can be found in the relative amounts of these alkaloids in crude opium; in fact, it has been reported that several cultivars of poppy straw have very low amounts of papaverine (Stranska et al., 2013). For what concerns morphine and codeine, the detection of these alkaloids is in accordance with other reports related to ancient vessels (Koschel, 1996; Linares et al., 2022); it can be postulated that even if morphine is relatively unstable, its detection in ancient vessels is possible due to its high concentration in the original content. This hypothesis is reinforced by the data provided by Nesmerak et al., who analyzed opium preparations aged more than 200 years and found that in some samples, morphine was still the main component (Nesměrák et al., 2019). In our samples, several factors may have contributed to the preservation of morphinan alkaloids and, especially, the fact that the vessels have been buried for a long period, with a low presence of oxygen.

In this regard, two hypotheses can be drawn for the four vessels (211, 317, 333, and 337) that showed no traces of alkaloids (<LOD): on one hand, it can be postulated that they were not used for the storage and/or consumption of opium and/or its preparations; on the other hand, it cannot be excluded that degradation may have occurred. In particular, the filter pot n. 337, which was selected because of the iconography which recalls the papaver plant, was extremely damaged both externally and internally. Conversely, it is extremely interesting that the vessel n. 265, already highlighted in Figure 1, because of the decorations that recall the poppy capsule, tested positive not only to thebaine but also to the less-stable morphine and codeine, suggesting the original presence of high amounts of opium alkaloids in this vessel. The jug 274, which showed decorations with the plant, tested positive only for thebaine.

**FIGURE 3:**
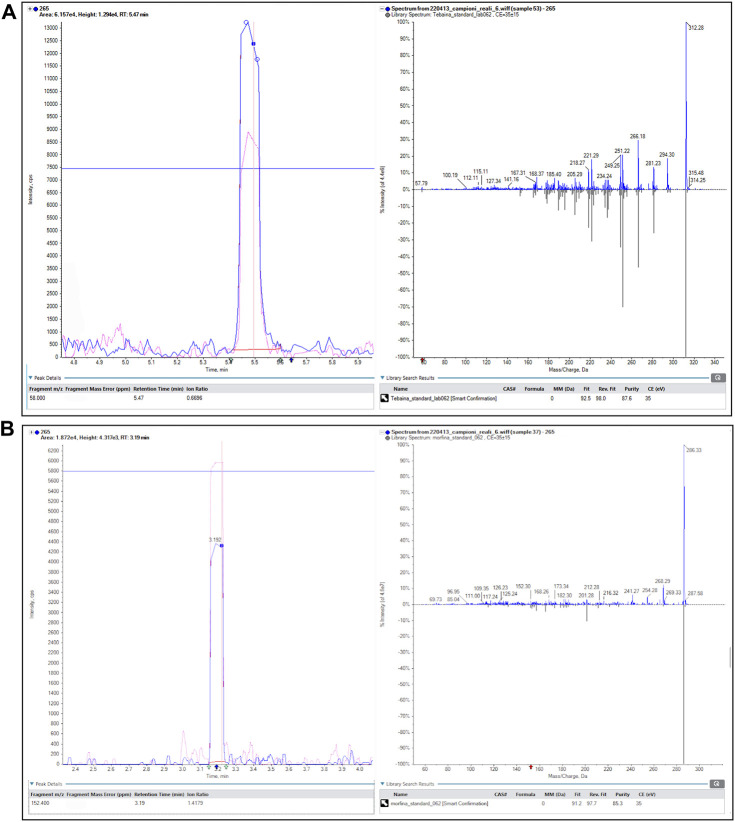
Example of a positive finding (vessel 265). The retention time of the peak, the presence of two overlapping precursor-to-product ion transitions, the ion ratio, and the mirror plot of the experimental MS/MS and library spectra are shown for thebaine **(A)** and morphine **(B)**.

The presence of the three alkaloids among some of the analyzed samples was confirmed by low- and high-resolution mass spectrometry; as an example, Figure 3 shows a positive finding of thebaine (a) and morphine (b).

For what concerns the different collected samples, it is very interesting to note that, generally, positive findings are common to all the samples collected in a pot, independent of the sampling strategy; similarly, no trace of alkaloids was detected in all the samples collected from the four vessels. These results suggest that scraping and swabbing provided comparable results; the latter sampling strategy is by far less diffused for organic residue analysis on archeological samples; however, considering the lower invasiveness, it could represent an interesting new approach for sample collection in an archeological context.

## 4 Conclusion

With the aim of verifying the presence of opioids in a selection of ancient vessels belonging to the Ceci-Macrini collection, suitable analytical methods were developed and validated.

Both targeted and untargeted analyses were performed to assess the presence of opium alkaloids, such as morphine, codeine, and thebaine. The obtained results, confirming the presence of opioids in 10 samples, shed new light on the hypothesis of opium usage in ancient Daunia. It is important to highlight that trace analyte detection was made possible by the developed analytical method, which is characterized by high sensitivity.

These results, which are included in a wide project joining analytical chemistry and archeology, are very promising, as they allowed assessing the presence of psychoactive substances in ancient samples. Furthermore, this study provided suitable analytical tools for further investigations on the same topic, with a good confidence level in the quality of the results. New analytical methods for sample preparation were presented and applied on real samples, and some interesting improvements were presented in these kinds of investigations. From an archeometrical perspective, a central result is the validation of sample collection by swabbing, and we demonstrated that it can be a valid alternative to scraping and it can be of particular relevance for archeological samples.

Future developments of the study will concern the inclusion of new target analytes such as scopolamine or other hallucinogenic substances; in fact, in the present analysis, extraction was specifically tailored for opiate detection so that other interesting molecules could be undetected. In addition, new Daunian vessels, arising from adequately documented archeological excavations, will be analyzed in future studies in order to confirm and validate the hypothesis of opium usage in ancient Daunia and, moreover, to make chronological and historical comparisons.

## Data Availability

The raw data supporting the conclusion of this article will be made available by the authors, without undue reservation.
